# Internal Jugular Central Venous Catheter Tip Migration: Patient and Procedural Factors

**DOI:** 10.3390/tomography8020083

**Published:** 2022-04-03

**Authors:** Tyler Smith, Claire Kaufman, Keith Quencer

**Affiliations:** 1 Department of Interventional Radiology, University of Utah, Salt Lake City, UT 84132, USA; 2 Department of Interventional Radiology Oregon Health and Sciences University, Portland, OR 97239, USA; kbquencer@gmail.com (C.K.); claire.kaufman@gmail.com (K.Q.)

**Keywords:** central venous catheter, catheter dysfunction, dialysis access

## Abstract

Background: The ideal central venous catheter (CVC) tip position placement is controversial, and CVCs do not remain in a fixed position after placement. This study evaluates both patient and procedural factors which may influence CVC tip migration and subsequent catheter dysfunction. Materials and Methods: This study evaluates CVC placements at a single institution. Patient age, gender, body mass index (BMI), catheter laterality, CVC type and indication for central venous access were recorded. Catheter tip location relative to the carina was measured at time of placement and removal utilizing supine fluoroscopic imaging. Patients’ electronic medical records were reviewed for evidence of catheter dysfunction. Statistical analysis was performed utilizing odds ratios and two tailed Student’s *t*-test. Results: 177 patients were included (101 female; mean age 55; mean BMI 29.2). Catheter types included 122 ports, 50 tunneled large bore central venous catheters (≥9 French), and 5 tunneled small bore central venous catheters (<9 French). 127 were right sided catheters, and 50 were left sided. Left sided CVCs had a mean cranial tip migration of 3.2 cm (standard deviation ±2.9 cm) compared to 0.8 cm (standard deviation ±1.9 cm) for right sided catheters (*p* = 0.000008). Catheters that migrated cranially by >2 cm had more than 7× greater risk of dysfunction compared to catheters that migrated ≤2 cm (odds ratio of 7.2; *p* = 0.0001). Left sided CVCs were significantly more likely to have >2 cm of cranial migration (odds ratio 6.9, 95% CI 3.4–14.2, *p* < 0.0001) and had a higher rate of dysfunction, likely due to this cranial migration (32% vs. 4.7%; *p* = 0.00001). Gender and BMI were not found to be associated with catheter dysfunction or an increased odds ratio of >2 cm cranial migration. Conclusions: Left-sided CVCs migrate an average of 2.4 cm cranially more than right-sided catheters. Additionally, when migration occurs, left-sided catheters are more likely to be dysfunctional. These suggest that lower initial placement may be beneficial in left-sided catheters.

## 1. Introduction

Central venous catheter (CVC) placement is a commonly performed procedure with estimates of approximately five million CVC insertions per year in the United States [1]. Various types of catheters are placed, including large bore catheters and small-bore catheters, peripherally inserted versus centrally inserted, cuffed, non-cuffed and totally implantable. The indications for placement of central venous catheters include hemodialysis, infusion of caustic medications such as chemotherapy or certain antibiotic medications, the administration of total parenteral nutrition (TPN), administration of intravenous hydration, difficult venous access requiring multiple blood draws, and central venous pressure monitoring [2].

Complications of CVC can be categorized as either early or late in relation to time of placement. Early complications are often related to procedural factors and include arterial puncture, transgression of the pleural space or mediastinum, or air embolism [3]. Late complications of CVC placement include infection, catheter fracture, catheter dysfunction or occlusion, and vessel thrombosis, stenosis or occlusion [3]. Superior vena cava (SVC) syndrome is one of the most serious delayed complications related to CVC placement causing great morbidity and mortality. SVC syndrome due to chronic indwelling catheter placement usually develops slowly, allowing for sufficient collateralization via the azygos and hemiazygos, lumbar veins, internal mammary to inferior epigastric and the thoracoepigastric vein [4]. However, it is important to be aware that when venous pressure rises about 50 mmHg, patients are at risk for cerebral edema, which can cause mental status changes, coma, or even lead to death due to cerebellar herniation [5].

The optimal positioning of the central venous catheter tip is of paramount importance to reduce late complications; however, the ideal tip position remains controversial [6,7,8,9]. A change in practice from CVC tip positioning in the SVC to tip positioning in the right atrium occurred in the late 1990s and 2000s as a response to papers showing a hemodialysis catheter whose tips at the right atrium work better for hemodialysis with lower rate of recirculation and ports whose tips are in the right atrium are less prone to dysfunction [6,10,11].

A central venous catheter whose tip terminates in the middle or upper third of the SVC increases the risk of SVC stenosis or thrombosis. This may be due to a variety of factors including vessel and endothelial trauma due to catheter tip contact with the vessel sidewall. This may lead to mechanical endothelial injury via denudation, focal hemorrhage, and lymphocyte infiltration. Catheters whose tips are in the upper third of the SVC are also prone to potential migration into the azygos vein [12,13,14,15,16,17]. Dialysis catheters have a jet of high flow out of the venous limb of the catheter and the arterial limb can suck up against the SVC wall. These factors cause endothelial injury which may lead to SVC stenosis or occlusion. Finally, the hypertonic or caustic medications which egress from the catheter tip may damage the thin endothelium of the SVC more easily compared to the thicker endocardium of the right atrium again leading to SVC stenosis.

If the CVC tip is placed too in the low right atrium or into the right ventricle, there is a potential risk of catheter dysfunction, arrhythmia, atrial mural thrombus, and, rarely, cardiac perforation [6,18,19,20]. Complications associated with central venous catheter dysfunction contribute to increased hospitalization and emergency department visits, and increases healthcare costs [21]. For example, one study estimated that vascular access dislodgement (a small subset of possible complications related to vascular access) accounts for at least 4.2 billion dollars in extra cost each year [21]. Despite the potential for complications, central venous catheters remain essential for some patients, such as patients with end stage renal disease requiring hemodialysis for whom an arteriovenous fistula or graft is not feasible due to stenosis, occlusion, or other anatomic factors, and they therefore depend on a central venous catheter.

Ports have a reported spontaneous catheter tip migration rate of 0.9–1.8% per year, however the mechanism of migration is not well understood [22]. A prior study found that catheter tip migration after chest wall CVC occurred in all patients with a trend towards lower rates of catheter malfunction in patients with the tip within the right atrium versus the superior vena cava [23].

While prior studies have confirmed that the tip of a CVC is not fixed, the relationship between the degree of CVC tip migration, laterality, gender, and BMI has not been fully described [6,23,24]. This retrospective study aims to investigate patient and procedural factors that could influence tip migration and subsequent dysfunction of central venous catheters to aid in optimal CVC placement.

## 2. Materials and Methods

Patient Selection:

Institutional review board approval was obtained prior to patient selection and chart review. This study was carried out in compliance with the Health Insurance Portability and Accountability Act (HIPAA). Patients were included who had fluoroscopic guided CVC placed by interventional radiology via the right or left internal jugular vein puncture between 2012 and 2021. Patients were excluded if they did not have supine fluoroscopic images showing the catheter tip, carina, and full length of the catheter images at both the time of placement and time of removal.

**Figure 1:**
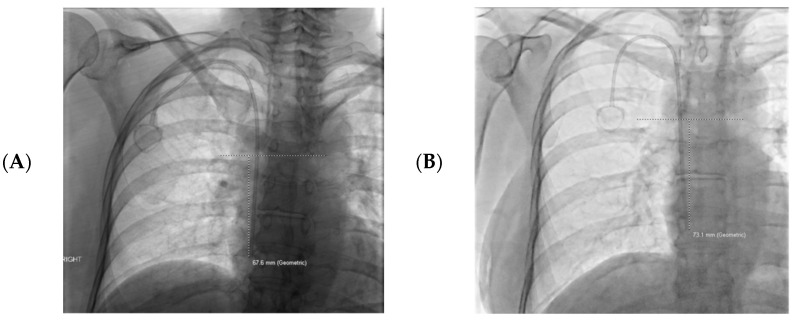
An example of catheter tip measurements obtained at the time of placement (**A**) and at the time of catheter removal (**B**). A line was made perpendicular to the carina to allow accurate measurement of the catheter tip distance from the carina. In this case the catheter advanced 3.7 cm caudally, and was recorded as −3.7 cm when calculating the data (positive numbers migrated cranially towards or beyond the carina).

Data Collection and Data Items:

Catheter tip migration was defined as any change in catheter tip position from placement to removal relative to the carina. Catheter migration towards or cranial to the carina was recorded as a positive number. Catheter migration caudal to the carina was recorded as a negative number. Measurements were performed by a single author utilizing a picture archive and communication system (PACS) measurement tool (Phillips, Amsterdam, Netherlands) (Figure 1). Supine fluoroscopic images were evaluated at time of placement and again at the time of removal. The measurements were recorded for comparison to determine, if present, the degree of catheter tip migration. Patient charts were reviewed for evidence of catheter dysfunction; this was defined as the inability to inject, aspirate or achieve adequate flow rates when being used for dialysis. Patient age, gender, catheter laterality, catheter type, and patient BMI at the time of placement were recorded.

**Figure 2:**
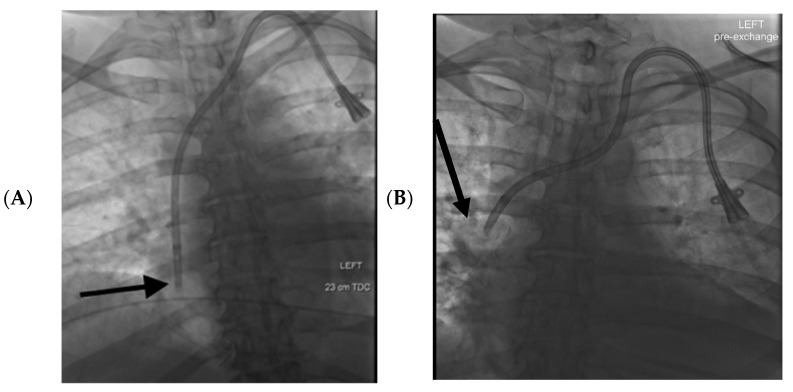
57-year-old female had a history of end stage renal disease with a left-sided tunneled dialysis catheter. (**A**): The initial supine fluoroscopic images at the time of catheter placement demonstrated the catheter tip within the mid right atrium (black arrow). (**B**): Two months later the patient presented with catheter dysfunction, and supine fluoroscopic interrogation demonstrated that the tip of the catheter had migrated 6 cm cranially to the upper SVC (black arrow).

Statistical Analysis:

Patients were grouped based on BMI, gender, type of catheter, catheter functionality, and laterality of CVC placement. Tunneled central venous catheters (excluding ports) were defined as small bore if less than 9 French, or large bore if greater than or equal to 9 French. The mean CVC tip migration and standard deviations were calculated (Figure 2). Odds ratios and a two-tailed Student’s *t*-test assuming unequal variance were performed. A *p*-value of 0.05 was used as a cutoff for significance. Comparison groups included the following: left versus right CVC placements, BMI ≥ 30 vs. < 30, male vs. female, tunneled ports vs. large bore tunneled CVC, and catheters with dysfunction vs. those without dysfunction.

Patient Characteristics:

Internal jugular central venous catheter insertions were reviewed in chronological order, and all patients who had fluoroscopic images at the time of placement and removal were included (total of 177 patients). Of the 177 included patients, 101 were female, 76 were male, and the mean age was 55 (range 21–87). The mean BMI was 29.2 (standard deviation ±7.6, range 16.75–66.5). Catheter types were as follows: 122 ports, 50 large bore tunneled CVC (lbTCVC), and five tunneled small-bore tunneled CVC (sbTCVC). 127 were right-sided catheters and 50 were left-sided (Table 1).

## 3. Results

Left-sided catheters had a mean catheter tip migration of 3.2 cm cranially (standard deviation ±2.9 cm) compared to 0.8 cm of cranial migration (standard deviation ±1.9 cm) for right-sided catheters (*p* < 0.001). Catheter tip migration >2 cm cranially was used as a cutoff for significance when calculating an odds ratio [25]. When comparing left- and right-sided catheters, we found that left-sided catheters had an increased odds ratio of greater than 2 cm cranial migration (odds ratio 6.9, 95% confidence interval 3.4–14.2, *p* = 0.000008) (Table 2).

Catheter dysfunction was observed in 22 patients (12%). Dysfunction was reported in 16 of 50 patients with left-sided catheters (32%), and six patients with right-sided catheters (4.7%) (*p* = 0.000001). Of the CVCs with reported dysfunction, 10 were ports, two were sbTCVC, and 10 lbTCVC. The mean cranial catheter tip migration for catheters with dysfunction was 4.3 cm (standard deviation ±3.0 cm). The mean cranial catheter tip migration for catheters without dysfunction was 1.0 cm (standard deviation ±2.2 cm) (*p* = 0.00006). Of the catheters that demonstrated dysfunction, 72% had a CVC tip migration of >2 cm cranially (16 of 22). The odds ratio for catheter dysfunction with cranial migration >2 cm was 7.2 (*p* = 0.0001, 95% confidence interval: 2.6–19.6).

Normal and underweight patients (BMI < 25) had a mean catheter tip migration of 1.4 cm cranially (standard deviation ±2.2 cm). Overweight patients (BMI between 25 and 29.9) had a mean catheter tip migration of 1.2 cm cranially (standard deviation ±2.9 cm). Obese patients (BMI ≥ 30) had a mean catheter tip migration of 1.7 cm cranially (standard deviation ±2.5 cm). There was no statistical difference found when comparing mean catheter tip migration for overweight and obese patients vs. patients with normal or low BMI (*p* = 0.8). There was no significant difference (*p* = 0.2) in catheter tip migration by gender (males 1.2 ± 2.5 cm, females 1.7 ± 2.4 cm). There was not a statistically significant increased risk of >2 cm cranial migration female vs. male patients (odds ratio 1.1, *p* = 0.7, 95% confidence interval: 0.6–2.1), ports vs. all other catheter types (odds ratio = 0.4, *p* = 0.007, 95% confidence interval: 0.2–0.8), or lbTCVC vs. all other catheter types (odds ratio = 1.8, *p* = 0.10, 95% confidence interval: 0.90–3.5). Ports had the least amount of cranial tip migration of (1.1 ± 2.0 cm), compared to lbTCVCs and sbTCVC (2.0 ± 3.2 cm and 4.9 ± 2.7 cm, respectively) which was statistically significant (*p* = 0.017; ports vs. all tunneled central venous catheters).

## 4. Discussion

CVC tip migration is commonly seen in clinical practice with one study from 1997 describing it in all patients; however, factors leading to its occurrence such as laterality, gender, BMI and catheter type are not well studied in the literature [23]. CVC tips which are too cranial (either because of initial placement or subsequent cranially migration) have a greater risk of catheter dysfunction, but can also lead to SVC stenosis, thrombosis, and contribute to the development of the morbid complication of SVC syndrome.

Factors such as gender, catheter type, patient BMI, and catheter laterality were investigated in this study because they have been proposed as possible factors which may contribute to catheter tip migration [26]. For example, reports have suggested that factors such as obesity or female gender may be associated with catheter tip migration due to changes in the position of soft tissues (such as breast tissue) when standing and pulling on the catheters, thereby shortening the length intravascularly [26]. Our results found no difference in catheter tip migration when comparing male vs. female (*p* = 0.2) and obese and overweight patients vs. all other patients (*p* = 0.8).

Catheter dysfunction was observed more frequently in CVCs that migrated cranially. The odds ratio for catheter dysfunction in patients whose catheters migrated >2 cm cranially was 7.2 (*p* = 0.0001, 95% confidence interval 2.6–19.6). This supports previously published literature, and is likely due to catheter contact with the walls of the SVC causing stenosis or thrombosis [12,13,14,15,16]. Previous studies have proposed that right-sided catheters are preferred to left-sided catheters because of a lower rate of dysfunction [25,27]. The course of the catheter from the right internal jugular vein to the SVC is straighter than other possible accesses, allowing for shorter catheters and better flow rates [27]. The results of this study support a right-sided catheter preference, as we found a 6.8× greater rate of catheter dysfunction in left-sided CVCs compared to right-sided ones (*p* = 0.0001).

This study found a significantly higher rate of cranial migration in catheters placed in the left internal jugular vein compared to catheters placed in the right internal jugular vein (left sided mean migration 3.2 cm cranial vs. right sided mean migration 0.8 cm cranial; *p* = 0.000008). Cranial migration being a risk factor for catheter dysfunction is supported by the fact that left-sided catheters with less than 2 cm of cranial migration have a low rate of dysfunction (5.8%), similar to the rate of right-sided catheter dysfunction (4.7). The difference in catheter dysfunction between left-sided catheters with less than 2 cm of cranial migration and right-sided catheters did not demonstrate a statistical difference (*p* = 0.42). One proposed mechanism for why left-sided catheters may be more prone to increased cranial migration is due to the longer and more tortuous course of the left brachiocephalic vein [28,29,30]. The left brachiocephalic vein is approximately 4.5 cm longer than the right brachiocephalic vein, taking an oblique caudal course to the level of the upper manubrium where it joins the right brachiocephalic vein to form the SVC [28,29,30]. On the contrary, not only is the right brachiocephalic vein shorter (approximately 2.5 cm in length) but has a straight caudal course and is contiguous with the SVC [28,29,30]. Therefore, left sided catheters are often 4–6 cm longer than right sided catheters. The predominant polymer of venous access catheters, thermoplastic polyurethanes, are known to soften at body temperature, which may lead to more catheter laxity and increased migration in longer catheters [31]. A combination of these factors may be contributing to cranial migration of the catheter tip, however more research is needed to better determine why left-sided catheters tend to have a greater degree of migration and dysfunction.

An analysis of CVC type found that ports may be less likely to demonstrate cranial migration compared to all other catheter types, with an odds ratio of 0.4, suggesting a possible protective effect (*p* = 0.007). This could be due to the internalization of the entire system; however, further studies are needed confirm this finding.

This study is unique, as other studies have reported catheter tip differences between supine placement imaging and subsequent upright imaging [32,33,34]. In this study, tip position was evaluated consistently in the supine position, both at the time of placement and removal, which would ideally control for changes in projection, differences in imaging modality and technique, and any shift in body soft tissues.

Limitations of this study include the retrospective design, data obtained from a single institution, non-randomization, and the limited number of left-sided catheters on sequential review (50 left vs. 127 right). Catheter dysfunction was likely higher than was found during this retrospective review, as patients may have received care elsewhere or dysfunction may have not been adequately documented in the clinical records. Differences in catheter size was not evaluated as a risk factor for catheter tip migration in this study. Other factors such as coughing, forceful flushing of catheters, or the vigorous use of arms may also contribute to catheter tip migration but are difficult to account for, and were not directly investigated in this study.

## 5. Conclusions

Left-sided CVC placement was correlated with significantly greater risk of catheter dysfunction and cranial tip migration. Lower initial placement of the catheter tip relative to the carina may be considered in select patients undergoing left-sided CVC placement. Ports had an overall lower rate of catheter tip migration compared to other types of CVCs. Other risk factors such as female gender and obesity were not associated with cranial migration of the catheter tip in this retrospective study. Further studies (including prospective studies) are needed to ensure the external validity of these results, and to investigate other factors such as catheter size on catheter tip migration.

## Figures and Tables

**Table 1:** Patient and catheter characteristics were reported as total number and percent. The total number of catheters placed was 177, with the majority being placed on the right side (72%). The most common catheter type was a port (69%). There was a slight majority of female patients (57%).

**Gender**	101 Female (57%)		76 Male (43%)	
**Laterality**	127 R (72%)		50 L (28%)	
**Catheter type**	122 Ports (69%)	50 lbTCVC (28%)		5 sbTCVC (3%)

lbTCVC = Large bore tunneled dialysis catheter; sbTCVC = Small bore tunneled central venous catheters.

**Table 2:** Mean migration, catheter dysfunction odds ratio, and odds ratio for >2 cm cranial migration for left vs. right sided CVCs.

	Left Sided CVC (*n* = 50)	Right Sided CVC (*n* = 127)	Statistical Analysis
Mean catheter tip migration	3.2 cm cranially	0.8 cm cranially	*p* = 0.000008 *
Catheter Dysfunction	16 (32%)	6 (4.7%)	Odds ratio for CVC dysfunction and >2 cm cranial tip migration: Odds ratio: 7.2 95% confidence interval 2.6–19.6 *p* = 0.0001
>2 cm catheter tip cranial migration	32 (64%)	26 (20.5%)	Odds ratio for >2 cm of cranial catheter tip migration in left vs. right CVCs Odds ratio: 6.9 95% confidence interval 3.4–14.2 *p* < 0.0001

*—Two Tailed Student’s *t*-test assuming unequal variance.

## Data Availability

Data for this study was obtained through retrospective review of patient medical records following IRB approval. All data presented has been anonymized.
